# Antioxidant Potential of Resveratrol as the Result of Radiation Exposition

**DOI:** 10.3390/antiox11112097

**Published:** 2022-10-24

**Authors:** Natalia Rosiak, Judyta Cielecka-Piontek, Robert Skibiński, Kornelia Lewandowska, Waldemar Bednarski, Przemysław Zalewski

**Affiliations:** 1 Department of Pharmacognosy, Poznan University of Medical Sciences, Rokietnicka 3, 60-806 Poznań, Poland; 2 Department of Medicinal Chemistry, Faculty of Pharmacy, Medical University of Lublin, Jaczewskiego 4, 20-090 Lublin, Poland; 3 Institute of Molecular Physics, Polish Academy of Sciences, Smoluchowskiego 17, 60-179 Poznań, Poland

**Keywords:** stilbenoid, electron paramagnetic resonance (EPR), FT-IR, electron beam radiation

## Abstract

The purpose of this study was to determine the effect of electron beam irradiation (EBI) at a dose of 25 kGy on the stability and antioxidant properties of resveratrol (RSV), a nutraceutical with clinically proven activity. The electron paramagnetic resonance (EPR) method was used to evaluate the concentration of free radicals after irradiation. Minor changes in chemical structure due to free radicals induced by EBI were confirmed by FTIR spectroscopy. HPLC and HPLC-MS analysis ruled out the appearance of degradation products after irradiation. In addition, HPLC analysis confirmed the absence of trans- to cis-resveratrol conversion. Changes in the antioxidant potential of RSV after irradiation were studied using DPPH, ABTS, CUPRAC, and FRAP techniques. It was confirmed that EBI favorably affected the antioxidant properties of tests based on the HAT mechanism (increase in DPPH and CUPRAC tests).

## 1. Introduction

Resveratrol (RSV), a natural compound, is a clinically proven nutraceutical [1,2,3]. It is found in many plants, including red grapes, mulberries, and peanuts. RSV’s valuable biological properties are due to its significant antioxidant potential [4]. The antioxidant potential of plant-derived structures (mainly flavonoids), including resveratrol, is important from the point of view of the possibility of neutralizing free radical forms that are formed during pathological processes (their excessive accumulation in the human body occurs in the process of treatment, whether during the use of pharmacotherapy or radiotherapy, and ultimately contributes to damage to macromolecules in the human body). Naturally occurring pathological processes that induce free radicals formation include neoplastic, neurodegenerative diseases (e.g., Alzheimer’s [5], Parkinson’s [6], Huntington’s [7]) or degenerative changes that occur with age (when intrinsic antioxidant mechanisms are less efficient). In addition, the antioxidant activity of resveratrol has been shown to protect tissues such as the liver and kidney from a various types of oxidative stress-induced damage [8]. Singh et al. described that the efficacy, safety, and pharmacokinetics of resveratrol have been documented in more than 244 clinical trials (data for the 2019 year) [2]. For example, resveratrol intake has been proven in clinical trials to have a positive effect on the treatment of Alzheimer’s disease (reduction of matrix metalloproteinase 9) [9,10], diabetes (lowering blood glucose levels, increasing insulin sensitivity) [11,12,13], non-alcoholic fatty liver disease [2,14] or cardiovascular disease (affects multiple molecular targets that are associated with cardioprotective effects) [15,16].

Electron radiation is widely used in many fields of knowledge. Of particular importance is the use of this technology to ensure the microbiological safety of food and drugs. One of the most important advantages of this method is that it is carried out at low temperature, which is especially important for heat-sensitive products. 

The literature reports on the chemical instability of many compounds of natural and synthetic origin after exposure to ionizing radiation [17,18,19,20]. There is also evidence that many substances are resistant to ionizing radiation [21,22,23,24]. Therefore, ionizing radiation can be used to obtain sterile forms of drugs (25 kGy) or to achieve adequate microbiological purity (15 kGy). The specified doses of ionizing radiation are justified by ISO 11137 standard, which defines them as the minimum necessary to achieve the specified microbiological purity/sterility requirement [25]. Many brands of nutraceuticals containing resveratrol are available, so it is important to check the stability and antioxidative potential of RSV after exposure to ionizing radiation [3,26].

The purpose of our study was to evaluate the radiostability of resveratrol following exposure to ionizing radiation (25 kGy) and to assess its antioxidant properties after exposure to electron beam irradiation. 

## 2. Materials and Methods

### 2.1. Materials 

Pure resveratrol (99%), potassium bromide (KBr), 2,2-Diphenyl-1-picrylhydrazyl (DPPH), Iron (III) chloride hexahydrate, 2,4,6-Tri(2-pyridyl)-s-triazine (TPTZ), ascorbic acid and neocuproine were supplied by Sigma Aldrich (St. Louis, MO, USA). Ammonium acetate (NH_4_Ac) and methanol were supplied by Chempur (Piekary Śląskie, Poland). Cupric chloride dihydrate, acetic acid (99.5%), ethanol (96%), sodium acetate trihydrate, and glacial acetic acid were supplied by POCH (Gliwice, Poland). Acetonitrile of an HPLC grade was supplied by Romil (Waterbeach, Cambridgeshire, UK). Direct-Q 3 UV system delivered ultrapure water. (Millipore, Molsheim, France, model Exil SA 67120). 

### 2.2. Irradiation

Resveratrol was irradiated with an electron beam (NIIEFA, St. Petersburg, Russia) with a dose of 25 kGy on behalf of the Radiation Sterilization Plant of Medical Devices and Allografts. Parameters: set dose 25 kGy, transporter 0.620 m∙min^−1^, set current 500 mA, energy 10 MeV, calibration factor 15.5, sampling 0.3 s.

### 2.3. Electron Paramagnetic Resonance (EPR) Spectroscopy

Free radicals detection was carried out at room temperature using a multi-frequency (S, X and Q-band) ELEXSYS 500 spectrometer (Bruker, Billerica, MA, USA). EPR spectra of resveratrol powder were recorded at X-band, using low microwave power (2 mW) to avoid line saturation. Due to the low signal-to-noise ratio for the samples, each spectrum was accumulated ten times. Free radicals concentration was determined by comparing the double integrated EPR spectra of resveratrol with a spin number standard (Al_2_O_3_:Cr^3+^). 

A sample and Al_2_O_3_:Cr^3+^ crystal with a known number of paramagnetic complexes (Cr^3+^ ions) were placed into the resonance cavity. EPR lines of Al_2_O_3_:Cr^3+^ were recorded below 2000 Gs, and above 4000 Gs. The ratio of the numbers obtained after the double integration of the standard line (EPR spectra were recorded as the first derivative of microwave absorption) and the tested sample allowed us to obtain the number of radicals in resveratrol. Before each line integration, the background of the spectrum was subtracted to obtain thee correct values. Both the integration and background correction of the spectra were carried out on the basic BRUKER Xepr 2.4b.28 program (Bruker, Billerica, MA, USA) used for recording and pre-processing of EPR spectra. Due to the low signal-to-noise ratio for radicals, we adjusted the spectra into single lines [27].

### 2.4. Fourier Transform Infrared Spectroscopy (FTIR)

The FTIR analysis of non-irradiated (0 kGy) and irradiated (25 kGy) resveratrol samples were performed. Absorption spectra were obtained under room temperature conditions on a Bruker Equinox 55 spectrometer (Bruker Optics, Ettlingen, Germany). Analyses were carried out in a KBr pellet (1 mg resveratrol sample and 200 mg KBr; diameter: 13 mm; pressure: 10 ton∙cm^−2^). The spectra were recorded in the wavelength region of 400–4000 cm^−1^ with 400 scans and a 4 cm^−1^ resolution. In all analyses, the pure KBr pellet was a blank sample. The obtained data were analyzed using the Origin 2021b software (OriginLab Corporation, Northampton, MA, USA).

### 2.5. Computation

The density functional theory (DFT) was used to optimize the molecular geometry of resveratrol. The geometries were fully optimized with B3LYP/6-311G (d,p) using Gaussian 09 software (Wallingford, CT, USA) [28]. GaussView program was used to visually inspect the normal modes [29].

### 2.6. HPLC and HPLC-MS Analysis

The Shimadzu Prominence-i LC-2030C HPLC instrument equipped with DAD detector was used in the study. The software was LabSolution DB/CS (version 6.50, Shimadzu, Kyoto, Japan). Solutions of irradiated and non-irradiated resveratrol were prepared in acetonitrile at a concentration of 0.4 mg∙mL^−1^. Solutions thus obtained were filtered through 0.45 μm syringe filters into 1.5 mL vials. Samples were measured on a Kinetex, C18, 100A, 100 × 2.1 mm column (Phenomenex, Torrance, CA, USA) with a particle sizes of 5 µm. The mobile phase was acetonitrile and 0.1% acetic acid (20:80 *v*/*v*) filtered through a 0.22 μm nylon membrane and ultrasonically degassed before use. The mobile phase flow rate was 1.0 mL∙min^−1^. The injection volume was 10 µL. Chromatograms were monitored at λ_max_ = 306 nm using the UV detector. Separation was performed at 40 °C, and the analysis time was 5 min per sample.

The Agilent high-resolution mass spectrometer (Q-TOF LC-MS system model) with electrospray ion source (ESI) and Infinity 1290 UHPLC liquid chromatography system consisting of a binary pump (G4220A), autosampler (G4226A), thermostat (G1330B), and DAD (G4212A) (Agilent Technologies, Santa Clara, CA, USA) were used. The MassHunter software was used for system control and data analysis. A Hibar RP-18e (2.1 × 50 mm, dp = 2 µm) column (Merck, Darmstadt, Germany) was used and isocratic elution by acetonitrile:water with 0.1% formic acid (10:90 *v*/*v*) was performed for 0.5 min. In the next step, a gradient elution was carried out to a composition ratio (60:40) within 9 min. The flow rate was 0.3 mL∙min^−1^, and thermostating at 35 °C was used. The main parameters were set as follows: MS: ESI—negative polarity, source temp 325 °C, drying gas 10 L∙min^−1^, nebulizer pressure 40 psig, capillary voltage 3500 V, fragmentor voltage 175 V, skimmer voltage 65 V, octopol RF 750 V. For spectral data recording, auto MS/MS mode was used with the range to mass: 90–1050 *m/z* and acquisition rate: 2 spectra∙s^−1^.

### 2.7. Antioxidant Assay 

Antioxidant activity was carried out using four methods: DPPH, ABTS, CUPRAC, and FRAP. The concentration ranges of resveratrol and vitamin C that were prepared for the study are shown in Table 1.

In a 96-well plate, the working solution and sample solution were added (6 replicates for each concentration). The plate was then wrapped with aluminum foil, shaken, and incubated at room temperature (DPPH/ABTS/CUPRAC) or 37 °C (FRAP). Color changes were read using a Multiskan GO UV reader (Thermo-Scientific, Waltham, MA, USA). The measurements were taken in duplicate. Ascorbic acid was used as a standard. 

The most important parameters of each method are shown in Table 2.

DPPH determination was performed according to the procedure given by Kikowska et al. [30]. A solution of the radical was prepared by dissolving 3.9 mg of DPPH in 50.0 mL of methanol. The solution was shaken in the dark for about 2 h. ABTS assay was performed according to the procedure outlined by Chanaj-Kaczmarek et al. [31]. Preparation of solutions for the assays: 7.0 mM ABTS in water and 2.45 mM aqueous potassium persulfate (1:1 *v*/*v*) were mixed. The solution was shaken in the dark for about 24 h. It was then diluted with deionized water until the absorbance reached ~0.77 (measured at 734 nm). CUPRAC assay was performed according to the procedure outlined by Özyürek et al. [32]. Preparation of CUPRAC solution: mixed neocuproine solution (7.5 × 10^−3^ M), 10.0 mM copper (II) chloride solution, ammonium acetate buffer (pH 7.0) (1:1:1 *v*/*v*). FRAP assay was performed according to the procedure outlined by Benzie et al. [33]. Preparation of test solutions: 25.0 mL of acetate buffer (pH = 3.6), 2.4 mL of TPTZ solution and 2.5 mL of 20 mM aqueous FeCl_3_ ∙ 6 H_2_O solution were mixed.

The degree of radical scavenging for DPPH and ABTS effects by the sample was calculated using the following formula:

(1) $$\mathrm{the}\;\mathrm{degree}\;\mathrm{of}\;\mathrm{radical}\;\mathrm{scavenging}\;(\%)=\frac{A_{0}-A_{i}}{A_{0}}∙100\%,$$

where $A_{0}$ is the absorbance of the control and $A_{i}$ is the absorbance of the sample.

The results of DPPH and ABTS effects are presented as a plot of %inhibition versus concentration. The results of CUPRAC and FRAP effects are presented as a plot of absorbance versus concentration.

The IC_50_ or IC_0.5_ value was determined from linear (Equation (2)) or polynomial (Equation (3)) regression analysis.

(2) y=ax+b

where *x* is the final concentrations of the sample, *y* is the inhibition ratios, and *a* and *b* are the coefficients.

(3) $$y=ax^{2}+bx+c$$

where *x* is the final concentrations of the sample, *y* is inhibition ratios, and *a*, *b*, *c* are the coefficients.

*X* (final sample concentration) for IC_50_ was calculated when *Y* in the regression equation was substituted with 50. For IC_0.5_, *Y* was substituted with 0.5.

## 3. Results

Evaluation of the radiostability of resveratrol in the solid state 50 h and 597 h (EPR) after exposure to electron beam irradiation (dose of 25 kGy) was carried out by using methods such as EPR, FTIR, HPLC, and HPLC-MS. Changes in the antioxidant properties of irradiated resveratrol were checked (50 h after exposition)) by DPPH, ABTS, CUPRAC, and FRAP assay.

### 3.1. Electron Paramagnetic Resonance (EPR)

**Figure 1:**
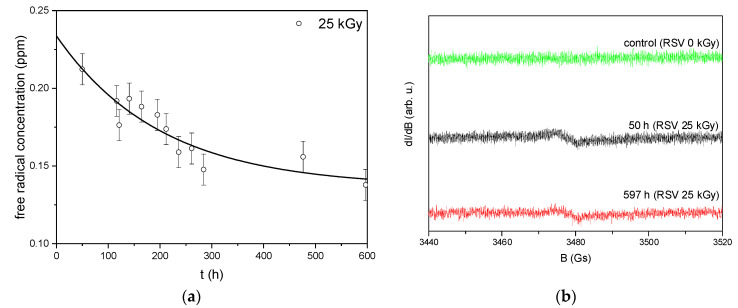
(**a**) Concentration of free radical vs. time after irradiation (25 kGy). The solid line is the approximation of Equation (4) to the experimental points; (**b**) EPR spectra of non-irradiated (green) and irradiated resveratrol recorded 50 h (black) and 597 h (red) after 25 kGy dose irradiation.

The EPR technique was used to evaluate changes of free radicals in solid-state resveratrol samples after irradiation (dose 25 kGy). Figure 1a shows the concentration of free radicals vs. time after irradiation calculated from EPR spectra for radiation dose 25 kGy. EPR spectra of non-irradiated and irradiated resveratrol recorded 50 and 597 h after irradiation are presented in Figure 1b. The EPR spectrum for the non-irradiated sample does not show any line from free radicals. In contrast, the irradiated sample exhibits a partial decrease of spectral intensity with respect to the time after irradiation. The decrease in free radical concentration vs. time for the irradiated sample can be described by the following equation [27]:

(4) $$C_{tot}\left(t\right)=C_{s}+C_{u}e^{-\frac{t}{T}}$$

where *C_tot_*(*t*) is the total concentration of free radicals determined at any time *t* after irradiation, *C_s_* is the concentration of stable radicals, *C_u_* is the concentration of unstable free radicals, t is time after irradiation, *T* is the mean lifetime of unstable radicals. After fitting the Equation (4) to the experimental points, the following values were obtained: *C_s_* = 0.14 ± 0.01 ppm, *C_u_* = 0.10 ± 0.01 ppm, and *T* = 202 ± 84 h.

### 3.2. Fourier Transform Infrared Spectroscopy (FTIR) Analysis

**Figure 2:**
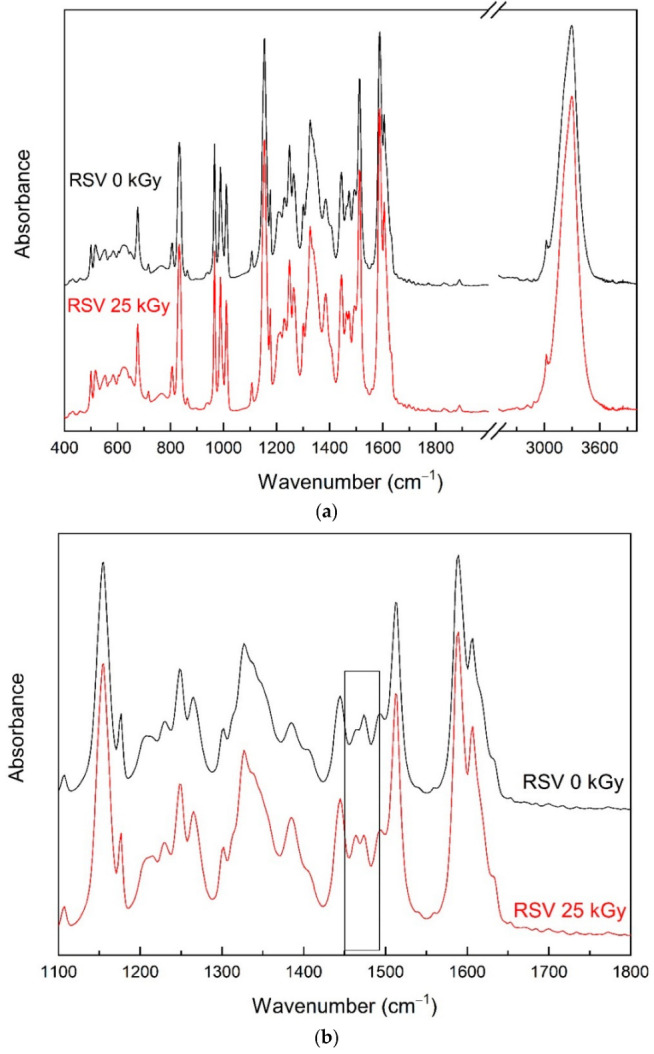
IR absorption spectra of non-irradiated (standard—black) and irradiated (red) resveratrol at room temperature: (**a**) range from 400 to 4000 cm^−1^; (**b**) range from 1100 to 1800 cm^−1^.

The assignment of resveratrol bands was made on the DFT study (Figure S1 and Table S1). All characteristic bands of RSV 0 kGy were observed after irradiation with a dose of 25 kGy (RSV 25 kGy, Figure 2a). However, the intensity of the band at about 1465 cm^−1^ (C-C stretching vibrations, C-O-H bending vibration and C-H rocking vibration in the hydroxyphenyl group) increased (Figure 2b). This may indicate minor oxidative damage to RSV molecules at the hydroxyphenyl group caused by free radicals, the presence of which was confirmed by EPR.

### 3.3. HPLC and HPLC-MS Analysis

HPLC and LC-MS confirmed that EBI did not cause conversion from trans- to cis-resveratrol. In addition, no degradation product was confirmed. It is likely that the changes caused by oxidative stress (sugessted in Section 3.2—FTIR analysis) were so small that they could not be recorded by these testing methods.

### 3.4. Antioxidant Properties

**Figure 3:**
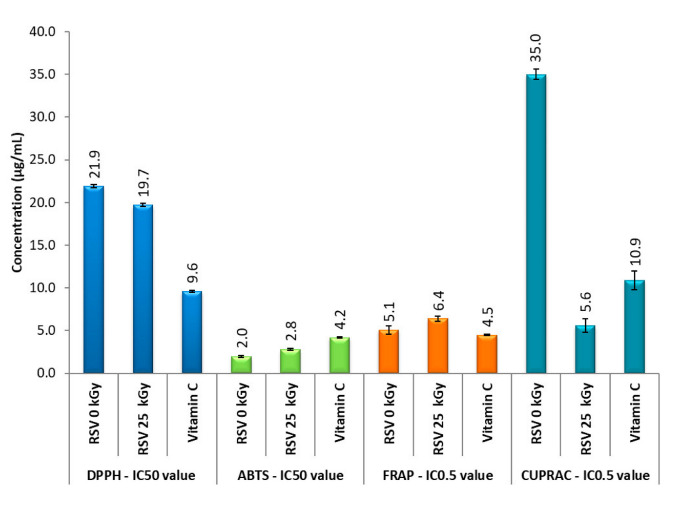
Summary of the results of antioxidant tests. Legend: X-axis—IC_50_ (DPPH and ABTS assay) or IC_0.5_ value (CUPRAC and FRAP assay), Y-axis—concentration of the sample, RSV 0 kGy—non-irradiated resveratrol, RSV 25 kGy—irradiated resveratrol.

Antioxidant properties of non-irradiated (RSV 0 kGy) and irradiated RSV (25 kGy) was tested using DPPH, ABTS, CUPRAC, and FRAP. The bar graphs (Figure 3) show the IC_50_ values for the DPPH and ABTS assay and the IC_0.5_ values for the CUPRAC and FRAP assay. Resveratrol (0 kGy) shows the best antioxidant properties for tests that rely on the SET mechanism (SET—transfer reaction of a single electron): ABTS (2 µg/mL) and FRAP (5.1 µg/mL). This is also indicated by theoretical calculations by Leopoldini et al. [34]. After irradiation of resveratrol with a dose of 25 kGy, we observe a slight decrease of antioxidant properties in ABTS and FRAP assays, and an increase of antioxidant properties in DPPH and CUPRAC assay, which are based on the HAT (HAT—hydrogen atom transfer) mechanism (Figure 3).

The changes in antioxidant activity of irradiated RSV may be attributed to the free radicals generated by electron beam radiation, which may cause oxidative damage to the RSV’s molecules. FT-IR analysis suggests that the changes involve the site in the structure of resveratrol that is most antioxidant active (position of OH groups in the para position) [35]. 

## 4. Discussion

The use of electron beam irradiation (EBI) has become an object of interest in the pharmaceutical and food industries due to the great potential of EBI bacteriocide and the fact that it causes less material degradation than other approaches. Nevertheless, it is confirmed that ionizing radiation (β-particles (electrons), γ-radiation, X-rays) may affect the biological properties of substances. The literature indicates the formation of degradation products [17,18,19], deterioration of antioxidant properties [36,37,38,39,40,41], improvement of antioxidant properties [42,43,44,45,46,47], and increase in anti-inflammatory properties [48] of substances exposed to radiation. Since the gas pedal accelerates the electron beam to near the speed of light (~99.999%c), the radiation transmits very high energy, affecting all material components in proportion to their electron contribution. During absorption of ionizing radiation, radicals are formed in the system, which, due to their reactive nature and short duration, cause complex reactions in the material. As a result, they can lead to changes in the structure, which can have a significant impact on the physicochemical properties of the material under study. 

Our research aimed to assess the radiostability of RSV, especially in the context of changes in its antioxidant properties after exposure to ionizing radiation. The studies conducted so far have focused on assessing the effects of the preventive action of resveratrol in living cells. Resveratrol, as with many other polyphenols, is a fundamental component of many nutraceuticals. Ensuring that the manufacturing process does not affect the properties of the product is essential to guarantee its effectiveness. To the best of our knowledge, no work has been published to date on the physicochemical and biological changes of resveratrol after exposure to electron beam irradiation (EBI). On the other hand, the assessment of after irradiation changes in resveratrol is important for the sake of ensuring the quality of resveratrol, when radiation sterilization is used as a compound stabilization technique. In our research, we used a dose of 25 kGy recommended by the pharmacopeia as appropriate to achieve microbiological stabilization and is large enough to exclude any changes in RSV during irradiation in living cells. The RSV post-sterilization changes were evaluated in the solid (EPR and FTIR) and after dissolving in water (HPLC, HPLC-MS).

EPR studies have shown that RSV is very resistant to the formation of radical damage caused by irradiation. The radiation dose of 25 kGy leads to the formation of only a small number of radical defects (not more than 0.24 ± 0.02 ppm), characterized by the EPR line with the spectroscopic splitting factor g = 2.0051 ± 0.0005 and linewidth ΔBpp = 5.9 ± 0.5 Gs. Most of the radicals, probably those close to the surface, are unstable and they decay when exposed to air particles. While the analysis of the bands of the infrared absorption spectrum of RSV carried out on the basis of a comparison with the theoretical spectrum (obtained as a result of calculations with the use of DFT) confirmed little changes in the hydroxyphenyl group. Bearing in mind that resveratrol requires dissolution in order to deliver it to the body, and the fact that in a dissolved form it is transported in body fluids, its stability in solutions was analyzed. HPLC-MS analysis of the chromatograms excludes the appearance of RSV impurities as radiolysis products. Moreover, the quantitative analysis of the peaks derived from the *trans* RSV isomer excludes its conversion to the cis form as a result of radiation transformation. It is likely that the changes in RSV’s structure (confirmed by FTIR analysis) caused by oxidative stress were so small that they could not be recorded by HPLC methods. 

It is known that the antioxidant activity of polyphenols is closely related to their structure [35]. Therefore, the next stage of the study was to check the effect of EBI on the antioxidant properties of resveratrol. 

RSV 0 kGy has a very different scavenging efficiency on DPPH and ABTS free radicals. It caused by different mechanisms. In the DPPH experiment, the hydrogen supply capacity of a compound determines the scavenging effect of free radicals (HAT mechanism), while ABTS experiment is determined by SET mechanism (single electron transfer) [35]. It is well known that phenolic groups stabilize a radical formed on phenolic carbon with their resonance structure. Due to the deprotonation of the hydroxyl groups present in resveratrol, we distinguish 3 acid dissociation constants: pKa_1_ = 8.8 (4-OH), pKa_2_ = 9.8 (3-OH or 5-OH), pKa_3_ = 11.4 (3-OH or 5-OH). According to the calculations by López et al. the para-4-hydroxy group is more acidic than the two meta-hydroxy groups [49]. Papuc et al. report that the OH group in the para position is the most antioxidant active site in RSV [35]. Test with CUPRAC reagent is carried out at pH = 7.0, which is close to the pH of physiological fluids. For this reason, this method is considered to be more advantageous compared to the FRAP test, which is carried out under alkaline conditions (pH = 3.6).

After irradiation of resveratrol with a dose of 25 kGy, we observed a slight decrease of antioxidant properties in the ABTS and FRAP assays and an increase of antioxidant properties in DPPH assay (a change of 2.2 µg/mL) and CUPRAC assay (a change of 29.4 µg/mL) (Figure 3). The changes in antioxidant activity of RSV 25 kGy may be probably attributed to the oxidative damage of RSV’s molecules, caused by the presence of free radicals. Their presence in the RSV 25 kGy sample was confirmed by EPR analysis. In addition, the FT-IR analysis suggests the changes in RSV’s structure concern in the most antioxidant active site of RSV: OH group in the para position. The antioxidant property study conducted clearly indicate that EBI enhanced the action of the HAT mechanism in the DPPH and CUPRAC test.

The obtained results of maintaining or improving the antioxidant properties of counseling resveratrol correspond with the results of other research groups [42,43,44,45,46,47]. It was confirmed that it is possible to increase the antioxidant potential of biologically active compounds as a result of their exposure to ionizing radiation. For example, Shah et al. [50] reported that after irradiation the DPPH scavenging ability of oat β-glucan increase with irradiation dose (0 kGy: 6%, 6 kGy: 14%, 10 kGy: 19% inhibition). Also, research by Khan et al. showed increased activity for β-D-glucan extracted from *Agaricus bisporus*. The doses of γ radiation they administered were 0–50 kGy, for which the inhibition activity was, respectively, 34.63–49.36% [51]. An increase in DPPH radical scavenging activity with an increase in the irradiation dose was also observed for bean starches [51], soybean [52], and green tea leaf extracts [53]. Slightly increased antioxidant activity was observed for 10–20 kGy of γ irradiation in the case of extracts from *Antrodia camphorata mycelia* [54]. Interesting results were obtained by Ahn et al. for irradiated phytic acid. Non-irradiated phytic acid did not show scavenging ability, whereas phytic acid irradiated at 20 kGy, showed significantly higher DPPH radical scavenging capacity than ascorbic acid at the 800 µM level [55]. On the other hand, there are reports indicating no change or deterioration of antioxidant properties after irradiation [36,37,38,39,40,41]. For example, Lampart-Szapa et al. reported that increased irradiation doses decreased the antioxidant effect of most lupin extracts [40]. Al-Kuraieef et al. also observed this effect in the case of the methanolic extract of thyme [39]. Other studies conducted for the cinnamon compound in the dose range of 5–25 kGy did not show any effect on the antioxidant activity [36].

## 5. Conclusions

Analysis of the structure of resveratrol exposed to electron beam radiation (25 kGy) allows us to indicate minor oxidative damage to resveratrol molecules. Importantly, these changes improved antioxidant properties of resveratrol based on the HAT mechanism. This knowledge can be helpful in the production of nutraceuticals containing resveratrol in their formulation.

## Figures and Tables

**Table 1:** Ranges of resveratrol and ascorbic acid concentrations used in studies of antioxidant properties.

Method	Solution of Resveratrol	Solution of Ascorbic Acid
DPPH assay	0.4–0.025 mg·mL^−1^	100−10 μg·mL^−1^
ABTS assay	0.2–0.005 mg·mL^−1^	100−10 μg·mL^−1^
CUPRAC assay	0.4–0.025 mg·mL^−1^	125−8 μg·mL^−1^
FRAP assay	0.2–0.025 mg·mL^−1^	300−100 μg·mL^−1^

**Table 2:** The most important parameters of DPPH, ABTS, CUPRAC, and FRAP activity.

Method	Sample Solution + Working Solution	Incubation	Measured
DPPH	25 µL + 175 µL	30 min reaction, 5 min: 600 rpm, 25 °C	517 nm
ABTS	10 µL + 200 µL	10 min reaction, 10 min: 600 rpm, 25 °C	734 nm
CUPRAC	50 µL + 150 µL	30 min reaction, 5 min: 600 rpm, 25 °C	450 nm
FRAP	25 µL + 175 µL	30 min reaction, 30 min: 100 rpm, 37 °C	593 nm

## Data Availability

The data are contained within the article and supplementary materials.

## Supplementary Materials

The following supporting information can be downloaded at: https://www.mdpi.com/article/10.3390/antiox11112097/s1, Figure S1. Calculation (black-DFT) and experimental (red) IR absorption spectra of resveratrol at room temperature; Table S1. Selected characteristic vibronic features of resveratrol theory with application of 6–31 G (d,p) basis and experiment bands of resveratrol. s-stretching, b-bending, r-rocking, oop-outside of the plane.

## References

[1] Berman A.Y., Motechin R.A., Wiesenfeld M.Y., Holz M.K. (2017). The therapeutic potential of resveratrol: A review of clinical trials. NPJ Precis. Oncol..

[2] Singh A.P., Singh R., Verma S.S., Rai V., Kaschula C.H., Maiti P., Gupta S.C. (2019). Health benefits of resveratrol: Evidence from clinical studies. Med. Res. Rev..

[3] Rossi D., Guerrini A., Bruni R., Brognara E., Borgatti M., Gambari R., Maietti S., Sacchetti G. (2012). Trans-resveratrol in nutraceuticals: Issues in retail quality and effectiveness. Molecules.

[4] Zhang L.-X., Li C.-X., Kakar M.U., Khan M.S., Wu P.-F., Amir R.M., Dai D.-F., Naveed M., Li Q.-Y., Saeed M. (2021). Resveratrol (RV): A pharmacological review and call for further research. Biomed. Pharmacother..

[5] Pan X., Zhu Y., Lin N., Zhang J., Ye Q., Huang H., Chen X. (2011). Microglial phagocytosis induced by fibrillar β-amyloid is attenuated by oligomeric β-amyloid: Implications for Alzheimer’s disease. Mol. Neurodegener..

[6] Sevcsik E., Trexler A.J., Dunn J.M., Rhoades E. (2011). Allostery in a Disordered Protein: Oxidative Modifications to α-Synuclein Act Distally To Regulate Membrane Binding. J. Am. Chem. Soc..

[7] Zhao W., Varghese M., Yemul S., Pan Y., Cheng A., Marano P., Hassan S., Vempati P., Chen F., Qian X. (2011). Peroxisome proliferator activator receptor gamma coactivator-1alpha (PGC-1α) improves motor performance and survival in a mouse model of amyotrophic lateral sclerosis. Mol. Neurodegener..

[8] Schmatz R., Perreira L.B., Stefanello N., Mazzanti C., Spanevello R., Gutierres J., Bagatini M., Martins C.C., Abdalla F.H., Daci da Silva Serres J. (2012). Effects of resveratrol on biomarkers of oxidative stress and on the activity of delta aminolevulinic acid dehydratase in liver and kidney of streptozotocin-induced diabetic rats. Biochimie.

[9] Moussa C., Hebron M., Huang X., Ahn J., Rissman R.A., Aisen P.S., Turner R.S. (2017). Resveratrol regulates neuro-inflammation and induces adaptive immunity in Alzheimer’s disease. J. Neuroinflamm..

[10] Turner R.S., Thomas R.G., Craft S., Van Dyck C.H., Mintzer J., Reynolds B.A., Brewer J.B., Rissman R.A., Raman R., Aisen P.S. (2015). A randomized, double-blind, placebo-controlled trial of resveratrol for Alzheimer disease. Neurology.

[11] Brasnyó P., Molnár G.A., Mohás M., Markó L., Laczy B., Cseh J., Mikolás E., Szijártó I.A., Mérei A., Halmai R. (2011). Resveratrol improves insulin sensitivity, reduces oxidative stress and activates the Akt pathway in type 2 diabetic patients. Br. J. Nutr..

[12] Thazhath S.S., Wu T., Bound M.J., Checklin H.L., Standfield S., Jones K.L., Horowitz M., Rayner C.K. (2016). Administration of resveratrol for 5 wk has no effect on glucagon-like peptide 1 secretion, gastric emptying, or glycemic control in type 2 diabetes: A randomized controlled trial. Am. J. Clin. Nutr..

[13] Bhatt J.K., Thomas S., Nanjan M.J. (2012). Resveratrol supplementation improves glycemic control in type 2 diabetes mellitus. Nutr. Res..

[14] Izzo C., Annunziata M., Melara G., Sciorio R., Dallio M., Masarone M., Federico A., Persico M. (2021). The role of resveratrol in liver disease: A comprehensive review from in vitro to clinical trials. Nutrients.

[15] Agarwal B., Campen M.J., Channell M.M., Wherry S.J., Varamini B., Davis J.G., Baur J.A., Smoliga J.M. (2013). Resveratrol for primary prevention of atherosclerosis: Clinical trial evidence for improved gene expression in vascular endothelium. Int. J. Cardiol..

[16] Magyar K., Halmosi R., Palfi A., Feher G., Czopf L., Fulop A., Battyany I., Sumegi B., Toth K., Szabados E. (2012). Cardioprotection by resveratrol: A human clinical trial in patients with stable coronary artery disease. Clin. Hemorheol. Microcirc..

[17] Ogrodowczyk M., Dettlaff K., Bednarski W., Ćwiertnia B., Stawny M., Spólnik G., Adamski J., Danikiewicz W. (2018). Radiodegradation of nadolol in the solid state and identification of its radiolysis products by UHPLC–MS method. Chem. Pap..

[18] Zalewski P., Rosiak N., Kilińska K., Skibiński R., Szymanowska D., Tykarska E., Piekara-Sady L., Lewandowska K., Miklaszewski A., Piontek J. (2020). The radiolytic studies of panipenem in the solid state. Acta Pol. Pharm. Drug Res..

[19] Ogrodowczyk M., Dettlaff K., Kachlicki P., Marciniec B. (2015). Identification of Radiodegradation Products of Acebutolol and Alprenolol by HPLC/MS/MS. J. AOAC Int..

[20] Zhuan R., Wang J. (2019). Degradation of sulfamethoxazole by ionizing radiation: Kinetics and implications of additives. Sci. Total Environ..

[21] Zalewski P., Skibiński R., Szymanowska-Powałowska D., Piotrowska H., Kozak M., Pietralik Z., Bednarski W., Cielecka-Piontek J. (2016). The radiolytic studies of cefpirome sulfate in the solid state. J. Pharm. Biomed. Anal..

[22] Janiaczyk M., Jelińska A., Woźniak-Braszak A., Bilski P., Popielarz-Brzezińska M., Wachowiak M., Baranowski M., Tomczak S., Ogrodowczyk M. (2022). Electron Beam Radiation as a Safe Method for the Sterilization of Aceclofenac and Diclofenac—The Usefulness of EPR and 1H-NMR Methods in Determination of Molecular Structure and Dynamics. Pharmaceutics.

[23] Kilińska K., Cielecka-Piontek J., Skibiński R., Szymanowska D., Miklaszewski A., Lewandowska K., Bednarski W., Mizera M., Tykarska E., Zalewski P. (2019). The Radiation Sterilization of Ertapenem Sodium in the Solid State. Molecules.

[24] Kilińska K., Cielecka-Piontek J., Skibiński R., Szymanowska D., Miklaszewski A., Bednarski W., Tykarska E., Stasiłowicz A., Zalewski P. (2018). The Radiostability of Meropenem Trihydrate in Solid State. Molecules.

[25] (2013). Sterilization of Health Care Products—Radiation—Part 2: Establishing the Sterilization Dose.

[26] Tomé-Carneiro J., Gonzálvez M., Larrosa M., Yáñez-Gascón M.J., García-Almagro F.J., Ruiz-Ros J.A., García-Conesa M.T., Tomás-Barberán F.A., Espín J.C. (2012). One-year consumption of a grape nutraceutical containing resveratrol improves the inflammatory and fibrinolytic status of patients in primary prevention of cardiovascular disease. Am. J. Cardiol..

[27] Mai V.C., Bednarski W., Borowiak-Sobkowiak B., Wilkaniec B., Samardakiewicz S., Morkunas I. (2013). Oxidative stress in pea seedling leaves in response to Acyrthosiphon pisum infestation. Phytochemistry.

[28] Frisch M.J., Trucks G.W., Schlegel H.B., Scuseria G.E., Robb M.A., Cheeseman J.R., Scalmani G., Barone V., Petersson G.A., Nakatsuji H. (2016). Gaussian 09, Revision C. 01.

[29] Dennington R., Keith T., Millam J. (2009). GaussView.

[30] Kikowska M.A., Chmielewska M., Włodarczyk A., Studzińska-Sroka E., Żuchowski J., Stochmal A., Kotwicka M., Thiem B. (2018). Effect of pentacyclic triterpenoids-rich callus extract of *Chaenomeles japonica* (Thunb.) Lindl. ex spach on viability, morphology, and proliferation of normal human skin fibroblasts. Molecules.

[31] Chanaj-Kaczmarek J., Wysocki M., Karachitos A., Wojcińska M., Bartosz G., Matławska I., Kmita H. (2015). Effects of plant extract antioxidative phenolic compounds on energetic status and viability of Saccharomyces cerevisiae cells undergoing oxidative stress. J. Funct. Foods.

[32] Özyürek M., Güçlü K., Apak R. (2011). The main and modified CUPRAC methods of antioxidant measurement. TrAC Trends Anal. Chem..

[33] Benzie I.F., Devaki M. (2018). The ferric reducing/antioxidant power (FRAP) assay for non-enzymatic antioxidant capacity: Concepts, procedures, limitations and applications. Measurement of Antioxidant Activity & Capacity: Recent Trends and Applications.

[34] Leopoldini M., Marino T., Russo N., Toscano M. (2004). Antioxidant properties of phenolic compounds: H-atom versus electron transfer mechanism. J. Phys. Chem. A.

[35] Hussein M.A. (2011). A convenient mechanism for the free radical scavenging activity of resveratrol. Int. J. Phytomed..

[36] Kitazuru E.R., Moreira A.V.B., Mancini-Filho J., Delincée H., Villavicencio A.L.C.H. (2004). Effects of irradiation on natural antioxidants of cinnamon (*Cinnamomum zeylanicum* N.). Radiat. Phys. Chem..

[37] Byun M.W., Son J.H., Yook H.S., Jo C., Kim D.H. (2002). Effect of gamma irradiation on the physiological activity of Korean soybean fermented foods, *Chungkookjang* and *Doenjang*. Radiat. Phys. Chem..

[38] Byun M.W., Yook H.S., Kim K.S., Chung C.K. (1999). Effects of gamma irradiation on physiological effectiveness of Korean medicinal herbs. Radiat. Phys. Chem..

[39] Al-Kuraieef A.N., Alshawi A.H. (2020). The effect of gamma irradiation on the essential oils and antioxidants in dried thyme. Int. J. Food Stud..

[40] Lampart-Szczapa E., Korczak J., Nogala-Kalucka M., Zawirska-Wojtasiak R. (2003). Antioxidant properties of lupin seed products. Food Chem..

[41] Ahn H.J., Kim J.H., Kim J.K., Kim D.H., Yook H.S., Byun M.W. (2005). Combined effects of irradiation and modified atmosphere packaging on minimally processed Chinese cabbage (*Brassica rapa* L.). Food Chem..

[42] Rajurkar N.S., Gaikwad K.N. (2012). Effect of gamma irradiation on antioxidant activity of *Amoora rohitaka*. J. Radioanal. Nucl. Chem..

[43] Hussein S.Z., Yusoff K.M., Makpol S., Yusof Y.A.M. (2011). Antioxidant Capacities and Total Phenolic Contents Increase with Gamma Irradiation in Two Types of Malaysian Honey. Molecules.

[44] Khalil M.I., Sulaiman S.A., Alam N., Moniruzzaman M., Bai’e S., Man C.N., Jamalullail S.M.S., Gan S.H. (2012). Gamma irradiation increases the antioxidant properties of tualang honey stored under different conditions. Molecules.

[45] Jung H.J., Park H.R., Jung U., Jo S.K. (2009). Radiolysis study of genistein in methanolic solution. Radiat. Phys. Chem..

[46] Choi J.I., Kim J.K., Srinivasan P., Kim J.H., Park H.J., Byun M.W., Lee J.W. (2009). Comparison of gamma ray and electron beam irradiation on extraction yield, morphological and antioxidant properties of polysaccharides from tamarind seed. Radiat. Phys. Chem..

[47] Lee H.J., Yoon M., Sung N.Y., Choi J. (2012). il Spirogyra varians mutant generated by high dose gamma-irradiation shows increased antioxidant properties. Radiat. Phys. Chem..

[48] Byun E.-B., Sung N.-Y., Park J.-N., Yang M.-S., Park S.-H., Byun E.-H. (2015). Gamma-irradiated resveratrol negatively regulates LPS-induced MAPK and NF-κB signaling through TLR4 in macrophages. Int. Immunopharmacol..

[49] López-Nicolás J.M., García-Carmona F. (2008). Aggregation state and p K a values of (E)-resveratrol as determined by fluorescence spectroscopy and UV-visible absorption. J. Agric. Food Chem..

[50] Shah A., Masoodi F.A., Gani A., Ashwar B.A. (2015). Effect of γ-irradiation on antioxidant and antiproliferative properties of oat β-glucan. Radiat. Phys. Chem..

[51] Khan A.A., Gani A., Shah A., Masoodi F.A., Hussain P.R., Wani I.A., Khanday F.A. (2015). Effect of γ-irradiation on structural, functional and antioxidant properties of β-glucan extracted from button mushroom (*Agaricus bisporus*). Innov. Food Sci. Emerg. Technol..

[52] Variyar P.S., Limaye A., Sharma A. (2004). Radiation-induced enhancement of antioxidant contents of soybean (*Glycine max Merrill*). J. Agric. Food Chem..

[53] Jo C., Son J.H., Lee H.J., Byun M.W. (2003). Irradiation application for color removal and purification of green tea leaves extract. Radiat. Phys. Chem..

[54] Huang S.J., Mau J.L. (2007). Antioxidant properties of methanolic extracts from *Antrodia camphorata* with various doses of γ-irradiation. Food Chem..

[55] Ahn H.J., Kim J.H., Jo C., Kim M.J., Byun M.W. (2004). Comparison of irradiated phytic acid and other antioxidants for antioxidant activity. Food Chem..

