# Supplementary material for: Antioxidant Potential of Resveratrol as the Result of Radiation Exposition
Source: Antioxidants (Basel). 2022 Oct 24;11(11):2097. doi: 10.3390/antiox11112097 (PMC9686677; doi:10.3390/antiox11112097)
Supplement: Supplementary file 1 [file antioxidants-11-02097-s001.zip › antioxidants-1933298-supplementary.pdf]

Antioxidant Potential of Resveratrol as the Result of Radiation Exposition

Natalia Rosiak [1], Judyta Cielecka-Piontek [1], Robert Skibiński [2], Kornelia Lewandowska [3], Waldemar Bednarski [3] and Przemysław Zalewski [1,*]

[1] Department of Pharmacognosy, Poznan University of Medical Sciences, Rokietnicka 3, 60-806 Poznań, Poland
[2] Department of Medicinal Chemistry, Medical University of Lublin, Jaczewskiego 4, 20-090 Lublin, Poland
[3] Institute of Molecular Physics, Polish Academy of Sciences, Smoluchowskiego 17, 60-179, Poznań, Poland

* Correspondence: pzalewski@ump.edu.pl

Fourier Transform Infrared Spectroscopy (FT-IR) analysis

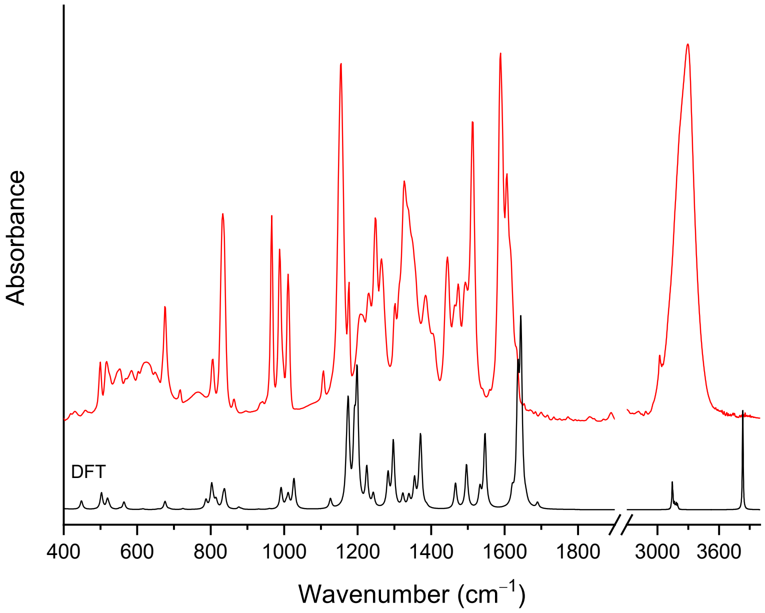

Figure S1. Calculation (black-DFT) and experimental (red) IR absorption spectra of resveratrol at room temperature.

Table S1. Selected characteristic vibronic features of resveratrol theory with application of 6-31G(d,p) basis and experiment bands of resveratrol. s-stretching, b-bending, r-rocking, oop-outside of the plane.

Calculation (cm^{-1})	Experimental (cm^{-1})	Band Assignment
504	499	Def. all molecule
520	515	Def. oop hydroxyphenyl group + def. dihydroxyphenyl group
565	550	Rocking dihydroxyphenyl group
677	676	C-H oop in dihydroxyphenyl group
788	768	C-H oop in dihydroxyphenyl group
803	805	C-H oop
839	833	C-H oop
877	863	Breathing hydroxyphenyl group
992	966	C-H oop in ethenyl group
1011	987	C-C s between dihydroxyphenyl and ethenyl groups + C-C-C b in dihydroxyphenyl group
1027	1010	C-O s in dihydroxyphenyl group + C-C s between dihydroxyphenyl and ethenyl groups + C-C-C b in dihydroxyphenyl group + rocking hydroxyphenyl group
1127	1107	C-H r in ethenyl group + C-O-H b
1175	1152	C-O s in dihydroxyphenyl group + C-H r in dihydroxyphenyl group + C-C s between dihydroxyphenyl and ethenyl groups
1199	1176	C-O-H b + C-H r
1225	1247	O-H r in dihydroxyphenyl group + C-H r in dihydroxyphenyl group
1243	1264	C-H r + C-C s between hydroxyphenyl and ethenyl groups
1284	1302	C-C s between hydroxyphenyl and ethenyl groups + C-H r
1298	1326	C-O s in hydroxyphenyl group + C-H r in hydroxyphenyl group
1356	1385	C-H r in ethenyl groups
1372	1444	C-O s in dihydroxyphenyl group + C-C s in dihydroxyphenyl group + C-O-H b in dihydroxyphenyl group C-H r in dihydroxyphenyl and ethenyl groups
1467	1465	C-C s + C-O-H b + C-H r in hydroxyphenyl group
1497	1474	C-O s + C-C s + C-O-H b in dihydroxyphenyl group
1534	1493	C-O s in dihydroxyphenyl group + C-C s between dihydroxyphenyl and ethenyl groups + C-H r in dihydroxyphenyl group
1547	1513	C-O s + C-H r in hydroxyphenyl group
1637	1588	C=C s + C-O-H b in dihydroxyphenyl and hydroxyphenyl groups
1644	1606	C=C s
1690	1636	C=C s in ethenyl group
3134	3023	C-H s
3203	3293	C-H s
3834	3400	O-H s

The IR absorption spectrum is characterized by many bands. Not taking the band at 3293 cm^{-1} into account, that is related to the stretching vibration of the C-H bonds, the bands at 1152 and 1588 cm^{-1} are the most intense in the spectrum. The first of them is mainly corresponding to the stretching vibration of the C-O bonds in dihydroxyphenyl group, but they have an additional component related to the rocking vibration of the C-H bonds in dihydroxyphenyl group, and stretching vibration of the C-C bond between dihydroxyphenyl and ethenyl groups. While the second band is associated

with the stretching vibration of the C=C bonds and bending vibration of the C-O-H bonds in dihydroxyphenyl and hydroxyphenyl groups. The bands related to the stretching vibration of the C-O bonds in dihydroxyphenyl group are also observed at 1010, 1352, 1474 and 1493 cm^{-1}. The first of them is also corresponding to the stretching vibration of the C-C bond between dihydroxyphenyl and ethenyl groups and bending vibration of the C-C-C bonds in dihydroxyphenyl group and rocking hydroxyphenyl group. The second have also additional components related to the stretching vibration of the C-C bond, bending vibration of the C-O-H bonds in dihydroxyphenyl group and rocking vibration of the C-H bonds in dihydroxyphenyl and ethenyl groups. And the band at 1474 cm^{-1} is also related to the stretching vibration of the C-C and bending vibration of the C-O-H bonds in dihydroxyphenyl group. The last band 1493 cm^{-1} is related to the stretching vibration of the C-C bonds between dihydroxyphenyl and ethenyl groups and rocking vibration of the C-H bonds in dihydroxyphenyl group too. The bands at 1210 and 1385 cm^{-1} are associated with the rocking vibration of the O-H and C-H in dihydroxyphenyl group and stretching vibration of the C-C, C-O and bending vibration of the C-O-H bonds in dihydroxyphenyl group, respectively. With the rocking vibration of the C-H and O-H bonds in dihydroxyphenyl group is also related the band at 1302 cm^{-1}. For the hydroxyphenyl group are characteristic the bands at 1264, 1444 and 1513 cm^{-1}. The first and last of them is associated with stretching vibration of the C-O bonds and rocking vibration of the C-H bonds. The second is corresponding to the stretching vibration of the C-C, bending vibration of the C-O-H and rocking vibration of the C-H bonds. In the IR absorption spectrum are also observed the bonds related to the stretching vibration of the C-C bond between hydroxyphenyl and ethenyl groups, which are located at 1229 and 1247 cm^{-1}. Whereas, the most characteristic bond for the ethenyl group is related to the stretching vibration of the C=C bond and is located at 1636 cm^{-1}. The band at 1606 cm^{-1} is associated with the stretching vibration of the C=C bonds in dihydroxyphenyl and hydroxyphenyl group. Above 3400 cm-1 is observed the band related to the stretching vibration of the O-H bond. In lower frequencies below 1000 cm^{-1} are mainly located the bands related to the deformation (rocking, breathing) of all molecule and vibration out of plane C-H bonds.
